# Involving people living with dementia in research: an accessible modified Delphi survey for core outcome set development

**DOI:** 10.1186/s13063-018-3069-6

**Published:** 2019-01-06

**Authors:** Hazel Morbey, Andrew J. E. Harding, Caroline Swarbrick, Faraz Ahmed, Ruth Elvish, John Keady, Paula R. Williamson, Siobhan T. Reilly

**Affiliations:** 1 Division of Health Research, Faculty of Health and Medicine, Furness College, Lancaster University, Lancaster, LA1 4YG UK; 2 Division of Nursing, Midwifery and Social Work, School of Health Sciences, Faculty of Biology, Medicine and Health, The University of Manchester, Manchester, UK; 3 MRC North West Hub for Trials Methodology Research, Department of Biostatistics, The University of Liverpool, Liverpool, UK

**Keywords:** Co-research, Dementia, Delphi methods, Inclusive research, Public involvement, Core outcome set, Neighbourhood

## Abstract

**Background:**

Recent recommendations promote the inclusion of people living with dementia beyond the role of ‘participant’ to involvement in all areas of the research process. This reflects shifts in dementia studies from ‘research on’ to ‘research with’ people living with the condition. In this paper, we describe the design process and features of a modified Delphi survey devised through consultation with people living with dementia.

**Methods:**

This article focusses on consultation with people living with dementia and care partners to design an accessible Delphi survey to facilitate participation in core outcome set development. We used the COINED model of co-research developed through the ESRC/NIHR Neighbourhoods and Dementia Study to structure consultation on three features of modified Delphi design. Consultation was achieved through 1:1 and group sessions with a total of 28 individuals (18 people living with dementia and seven care partners).

**Results:**

A flexible, responsive and adaptive approach to ongoing consultation with people living with dementia and care partners through 1:1 face-to-face sessions facilitated: (1) the development of a 3-point non-categorical importance scale; (2) the translation of 54 outcome areas into ‘accessible statements’ for a two-round Delphi survey administered to five stakeholder groups (people living with dementia, care partners, health and social care professionals, policy-makers and researchers); and (3) the delivery of a Delphi survey. These features of core outcome set development facilitated the involvement of people living with dementia in study design and as research participants in the data collection phase.

**Conclusions:**

Involvement of people living with dementia as a key stakeholder group is not reflected in studies using Delphi survey methods for core outcome set development. Time, resources, researcher expertise and support, underpinned through targeted funding facilitate meaningful and productive inclusive approaches, now an expectation of dementia research.

**Trial registration:**

The study is registered on the COMET Initiative.

## Background

There has been a notable shift in the research agenda in dementia studies from ‘research on’ to ‘research with’ people living with the condition [1, 2]. Dementia activists and groups, such as Kate Swaffer, the European Group of People with Dementia and the Dementia Engagement and Empowerment Project (DEEP), have sought to rebalance the priorities of the academic, health and social care communities with that of the lived experience – ‘nothing about us without us’ as the maxim proclaims [3, 4]. A recent position paper published by Alzheimer Europe [5] has put forward a set of recommendations that promotes the inclusion of people living with dementia beyond the realm of ‘participant’ to involvement in all areas of the research process in ways that are personally meaningful and which takes place within mutually respectful research relationships.

It has been suggested that development of optimal and varied methodologies in research are the keys necessary to facilitate the participation of older people in research [6]. In dementia studies, the CO-research INvolvement and Engagement in Dementia (COINED) model [7] is one example of such a facilitative approach and it was developed alongside people living with dementia as a framework for the involvement of people living with dementia as ‘co-researchers’. The term ‘co-researcher’ reflects collaborative, co-operative and community-based partnership between groups of people living with dementia, academic researchers and service providers. The COINED model forms part of the work of the 5-year ESRC/NIHR Neighbourhoods and Dementia Study (henceforth the Neighbourhoods Study), funded in the United Kingdom, under the first Prime Minister’s Challenge on Dementia [8]. Underpinned by the values of the Scottish Dementia Working Group’s core principles [9] and INVOLVE’s [10] user-involvement research brief, the COINED model outlines ways in which people living with dementia have identified how they would like to take part in the Neighbourhoods Study (and beyond) including roles in setting research priorities, involvement in the data collection processes, analysis, data collection and dissemination. In this paper we apply INVOLVE’s [10] definitions when using the following terms: *Participation* (where people take part in a research study); *Involvement* (where members of the public are actively involved in research projects and in research organisations); *Consultation* (when members of the public are asked for their views and these views are used to inform decision-making).

By adopting the framework of the COINED model, this article illustrates the ways in which people living with dementia were involved in Work Programme 3 attached to the Neighbourhoods Study and the approaches to involvement that were necessary to enable participation. The key aim of the study is to establish an agreed standardised core outcome set (COS) for use when evaluating non-pharmacological health and social care interventions for people living with dementia at home in their neighbourhoods [11]. Before the participatory approach is described further, we briefly outline the meaning and scope of a COS.

### Involving people living with dementia in COS research design

The Core Outcome Measures in Effectiveness Trials (COMET) Initiative website describes a COS as a set of outcomes that should be measured and reported, as a minimum, in all clinical trials, and, where appropriate, clinical audit or research on interventions other than randomised trials, of a specific condition. However, existing measures may not include outcomes that are of importance to people living with dementia, whose perspectives are often not represented [12–15] or are represented poorly [16, 17]. With approximately two thirds of people with dementia living at home, existing studies may not meaningfully reflect outcome measures of importance to people with dementia living at home. Through the application of the Neighbourhood Study's COINED model, the current study significantly contributes to this deficit by both privileging the outcome priorities of people living with dementia, and through the novel design of inclusive and participatory research methods employed to identify these outcomes.

Work Programme 3 of the Neighbourhoods Study applies COMET guidance for the development of COSs [18]. It includes a four-phase study design using a mixed-methods approach, to elicit outcomes of importance to people with dementia living at home, for community-based non-pharmacological interventions [11]. The Delphi method is increasingly recognised as a rigorous and robust approach to the development of COSs [16]. In addition, recent reviews have highlighted the need to develop strategies that use qualitative methods as a means to consult with key stakeholders to inform the design of COS research tools [16, 19]. More specifically, the use of qualitative methods to inform the design of Delphi surveys is being increasingly regarded as good practice [20].

Our mixed-methods study includes: a review of literature on reported dementia outcomes; qualitative methods data collection – focus groups/interviews with key stakeholders (people living with dementia; care partners; health and social care professionals; policy-makers; researchers); a modified Delphi process and consensus workshop with key stakeholders; a systematic review (to match existing outcome measures to core outcomes); and a stated preference survey (see full study published protocol [11]). ‘Care partners’ is the term selected by involvement groups in Work Programme 1 of the Neighbourhoods Study, to refer to those who look after, support and care for someone living with dementia, in a non-professional, non-paid capacity. This may be a family member, friend or neighbour. Consultation with 18 people living with dementia and seven care partners in a co-research capacity informed the design of survey methods, which are described in this paper. We report below on key areas of consultation that resulted in the development of a two-round modified Delphi survey.

### Ethics approvals

Full ethics approval was obtained from Lancaster University Research Ethics Committee and NHS Research Ethics Committee number 15/WA/0260 (17 July 2015). Consultation with people living with dementia for this study conformed with ethical practice principles set out in the study protocol and approval documentation to support inclusion and participation in dementia research [5].

## Methods

### Co-research consultation for modified Delphi survey design

Core outcome set development that uses literature review alone to identify outcomes is limited and cannot meaningfully reflect and embed public priorities in research. The Delphi method is increasingly used in COS studies and variation in the approach and critique is evident in relevant literature [20–22]. Adaptation to the Delphi survey method is necessary so as not to exclude key stakeholder groups from participating. Without adaptation, given a complex, multi-sequential process, the research team anticipated the potential exclusion of many people living with dementia in core outcome development. This was confirmed in consultation with people living with dementia, and reflected the outcome of the participation of people living with dementia in a previous Delphi survey which found ‘… in its traditional format is not feasible.’ [24:14]. For the current study, adaptation of a two-round Delphi method maximised inclusion of people living with dementia. We used the COINED model to identify points of potential co-researcher involvement and developed a structure for public involvement through consultation on different components of the survey design. We consulted individuals from three dementia groups with which we had connections through the Neighbourhoods Study partner organisations, or existing co-researcher relationships. We report on a mix of approaches used for co-research consultation on three key features of the Delphi survey design:

1. Accessible Delphi outcome items
2. Outcome item rating scale
3. Round-2 Delphi survey interface

### Sample: people living with dementia and care partners participating in consultation sessions

A total of 18 people living with dementia and seven care partners were consulted to gain feedback on Delphi survey design (Table 1). Care partners either accompanied as spouses of people living with dementia, or were previous care partners to people living with dementia either deceased or recently resident in care home facilities. Where one-to-one consultations included a person living with dementia and a care partner, both provided feedback through discussion.

**Table 1:** Stakeholders consulted on modified Delphi survey design

Stakeholder group	Individual 1:1 consultations	Group consultations		Total number of stakeholders consulted
		Number of groups	Number of participants	
People living with dementia (Formal or self-diagnosed dementia and living at home).	2	3	Group 1: 5	18
			Group 2: 5	
			Group 3: 6	
People living with dementia and care partner (Formal or self-diagnosed dementia and living at home).	3			3
Care partners (Current or previous experiences of providing care for a person living with dementia).	1	2	Group 1: 4	7
			Group 2: 2	

### Co-research consultation on three key features of a modified Delphi survey design

To facilitate the inclusion of people living with dementia in the COS we focussed on three specific aspects of Delphi survey design.

#### Modified design feature 1: Accessible Delphi outcome items

Through qualitative methods and literature review of non-pharmacological and community-based interventions or programmes for people living with dementia, 169 outcomes were identified. A further paper (in development) presents both the literature review outcomes together with the qualitative data identified outcomes, and discusses the synthesis of these in order to form a 54-item ‘long list’ of outcomes to be scored in a two-round Delphi survey. Each of the 54 outcome items were translated into ‘accessible statements’ to be scored using the 3-point scale outlined above, through a process of consultation and series of workshops. Consultation took place with people living with dementia and care partners in different settings, including in the community at dementia group venues and in people’s homes. People from these three stakeholder groups were not previously known to the research team, becoming involved in the study via local dementia networks or through attending dementia groups; for example, memory cafés, visited by the researchers. We also held eight developmental workshops, each with between four and six dementia specialist clinicians and researchers, to construct the ‘long list’ of outcomes. Participants in these workshops had a range of health and social care research backgrounds, including clinical and care-giving experience [11].

**Fig. 1:**
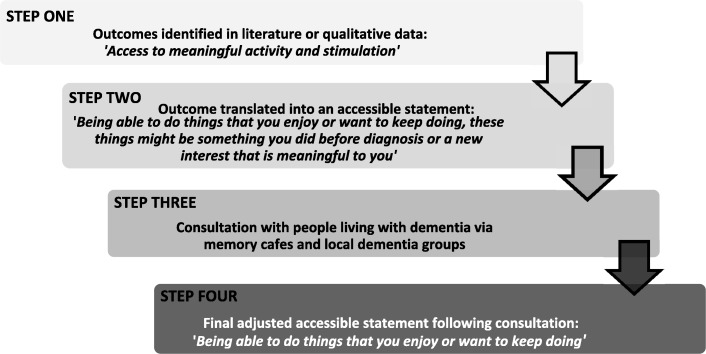
Development steps for translation of outcomes into accessible statements

We applied a four-step process to translate outcomes into descriptions that in turn represented accessible statements. Figure 1 outlines this process and uses one outcome, ‘meaningful activity’, as illustration. *Step 1*: outcome/s developed into a descriptive item (e.g. access to meaningful activity and stimulation); *Step 2*: descriptive statements written using simple, clear, non-technical and concise wording. In addition, an example was provided for each item (e.g. ‘Being able to do things that you enjoy or want to keep doing’); *Step 3*: descriptive statements were translated into ‘accessible statements’ by the research team. This involved initial feedback on a smaller number of items from the Neighbourhoods Study co-research lead and people living with dementia from dementia groups, following which the remainder of the accessible statements were devised; and *Step 4*: consultation feedback on all accessible statements and subsequent amendments finalised.

We devised a structure for co-researcher consultation, which included different approaches (one-to-one and group), and input from different stakeholders: people living with dementia, care partners and clinicians working in the area of dementia care or research.

#### Modified design feature 2: Outcome Item Rating Scale

The ‘Grading of Recommendations, Assessment, Development and Evaluation’ scale uses a 9-point scale (1–9) to grade evidence [23]. The advantage of having a 9-point scale over 5 points or less is that a 9-point scale gives greater scoring choice – i.e. respondents have more options/degree of agreement to select, which can result in greater diversity of responses/ratings. Some authors have reported that use of the Delphi method with people living with dementia indicates that even a 5-point scale would likely be unsuccessful [24]. This raises two main concerns: (1) is there a lower Delphi scale configuration that can be successful and (2) will there be enough diversity/variance in responses across the stakeholders?

**Fig. 2:**
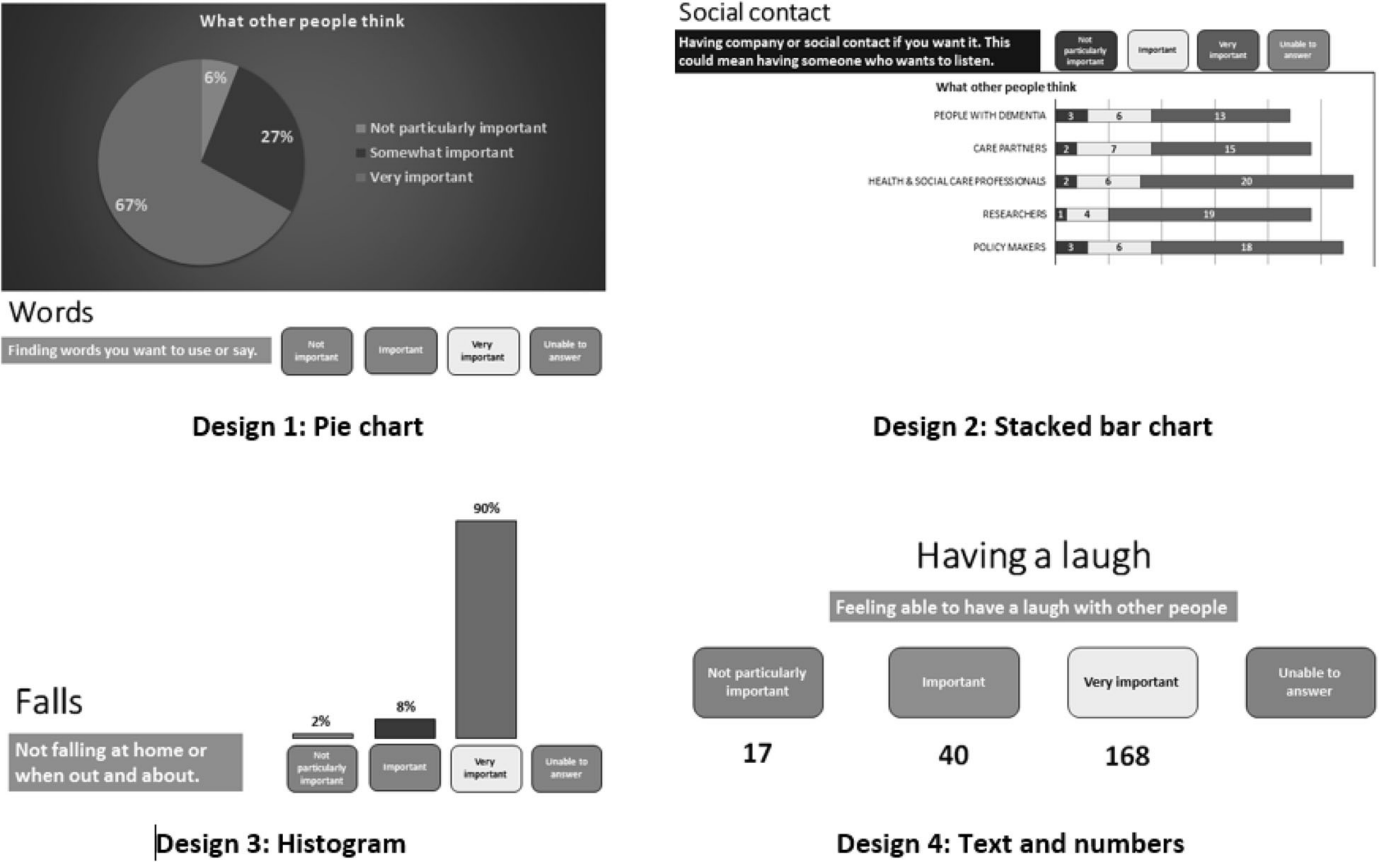
Examples of designs for presenting round-2 Delphi survey data

We designed and gained feedback on a 3-point scoring scale appropriate for use by people living with dementia and the other stakeholder groups: ‘Not particularly important’, ‘Important’ and ‘Very important’ (as seen in Fig. 2). Outcome items extracted from a literature review and identified through qualitative data collection were presented singularly on A4 paper sheets with a colour version of the scale in three boxes. Individuals scored the outcomes by circling the box that best reflected how important the item was to them. Researchers facilitated discussion, asked specific questions, and recorded comments in written note form.

#### Modified design feature 3: Round-2 Delphi survey interface

The Delphi process involves participants scoring items in a number of sequential survey rounds. An initial first round of scoring is completed, and this is followed by further round/s in which participants receive a summary of previous round scores for stakeholder groups (in graph or table form, or simple statistics such as mean or median) as well as a reminder of their own previous score. Participants are invited to reflect on their own score, together with the group scores, before scoring again. In so doing, participants can either change their score or retain their original score. Through consultation with people living with dementia, we sought feedback on design features for round 2 (R2) of the Delphi survey. This involved constructing different sample versions of round 1 (R1) data to be presented at R2 for subsequent scoring, again using the 3-point scale described above. Four design examples were presented at consultation meetings, these included: (1) Pie chart; (2) Stacked bar chart; (3) Histogram; and (4) Text with numbers (Fig. 2). Verbal and non-verbal responses to the visual features of design were recorded, including comments on coherence and comprehensiveness of content and instructions.

## Results

Each consultation session required specific planning to adapt an approach that was the most appropriate for the individual or group. During consultation sessions, adaptations were made by researchers as appropriate. Thus, during one session with a person living with dementia and their care partner, it became apparent very early on in the consultation that visuospatial abilities influenced what information was accessible. The researchers adjusted how they presented the accessible statements by reading some of these with the person living with dementia to clarify meaning, or they stood back when the care partner did so. In other group consultation sessions, individuals would read the accessible statements first before discussing and giving feedback. Thus, it became clear which R2 Delphi survey design examples were problematic for people with visuospatial challenges, and in other consultation sessions, discussion about the four designs identified relative strengths and weaknesses of each design sample.

### Features of Delphi design modification

#### Outcome Item 3-point Rating Scale

Consultation established that people living with dementia could make a selection on the 3-point scale in response to reading an accessible statement. In adapting a rating scale for use in our modified Delphi survey we understood it to be inadvisable to use a scale that accommodated extremes and would indicate categorical unimportance [24]; for example, at the negative end of the scale, using ‘Strongly disagree’ or ‘Not important’. For people living with dementia we believed it would be unlikely that outcomes would be categorically ‘Not important’, given that outcome areas had been extracted from Phase 1 qualitative data on areas of importance. Rather, it is preferable to ask participants to consider which areas are of less or more importance, thus acknowledging that all outcome categories may have some importance.

Our rating scale design took account of guidance that had developed appropriate ways to present materials to people living with dementia [25–27], and included consideration of factors such as: use of colour; minimal, clear wording; page layout and word placement; font; point size; page orientation; use of symbols and/or words; language. We anticipated that despite devising a non-categorical importance scale, there was potential for survey items to be predominately scored as ‘Very important’ given the origins of survey items from qualitative data in which people living with dementia described what was of importance to them. However, we found that differentiation was made between the scale scores, and that the two stakeholder groups differentiated in their answers, i.e. some people living with dementia and care partners who undertook the consultation together scored items the same, while others selected different scores from each other. This suggested that there was still variation/difference between the stakeholders using a scale with a lower number of points.

#### Accessible Delphi outcome items

We gained feedback on all 54 accessible statements through a combination of group and one-to-one consultation sessions, singularly with people living with dementia or with people living with dementia and care partners together. As simple and clear a design as possible was particularly important, with suggestions that words be kept to a minimum and as simple as possible. Jargon was replaced and font, point size, and the use of colour were identified as important, e.g. text highlighted with a yellow background helped the accessible statements to stand out from the text of the rating scale, which had a background colour of orange. People commented that word placement on the page and proximity of text facilitated understanding. It was proposed that study background information should be limited because this was not relevant to the immediate focus on the rating process, and was available in separate study information sheets.

Feedback on the use of examples to illustrate accessible statements was mixed. Some people commented that examples were helpful to orientate participants to an area. However, some suggested that examples could be restrictive and that accessible statements were better left open for individuals to reflect on meaning. Through team reviews of these detailed consultation discussions, it was felt that people living with dementia related to and recognised the areas represented by accessible statements, and that the intended depiction (of an outcome area) had been the understood depiction (area of lived experience). As a result, examples were limited to accessible statements where this had been described as being helpful, by people living with dementia. Thus, 17 of the 54 accessible statements included examples, illustrated by: ‘Being able to carry out more complicated activities … For example, cooking, shopping and managing money or medications’.

Two final feedback areas related to positive/negative emphases, and the sequence that accessible statements appeared in the survey. Of the total 54 statements, 48 were positively phrased (e.g. ‘Being free from frustration most of the time is ...’) and six had a negative emphasis (e.g. ‘Not asking the same questions again and again is ...’). The latter were phrased in this way because it proved difficult to re-word with a positive emphasis. Feedback suggested that it was acceptable to include negative items, as these were valid concerns for people living with dementia. In terms of sequence of accessible statements, comments suggested that it would be important to ensure that participants did not finish the survey scoring a negatively worded statement. Table 2 provides three examples of changes made to accessible statements following consultation. Importantly, accessible statements developed through consultation were used for all stakeholders, thus achieving consistency across all groups while ensuring accessibility for people living with dementia.

**Table 2:** Changes made to accessible statements following consultation with people living with dementia and care partners

Pre consultation		Post consultation	
Outcome item	Accessible statement	Outcome item	Accessible statement
Working with numbers	Difficulties with numbers; for example, using bus timetables	Numbers	Working with numbers
Language/word finding	Difficulties with language; for example, finding the word you want to say	Words	Finding words you want to use or say
Fear of deterioration	Fear of dementia symptoms getting worse	Dementia getting worse	Fear of dementia getting worse

#### Round 2 R2 Delphi survey interface

A Delphi survey is a sequential, multi-staged process of scoring separate survey rounds, where reflection, contrasting multiple stakeholder scores, retention of layers of information, decision-making and potential for changing a score are required in order to select a final R2 score. Feedback when testing elements of this multi-sequential process confirmed that it would be inappropriate to invite people living with dementia to take part in this approach. For example, it was apparent that the visual representation of R1 scores for each stakeholder group (Fig. 2), and the reflection process for a person living with dementia, proved confusing. Despite having presented minimal information with visual facilitators, e.g. colours, varied data display (bar/pie/histograms) and spacing, feedback on the array of information from one person with visuospatial difficulties, was that all the examples were inaccessible. The visual designs, numbers and text, could either not be seen or were unrecognisable. Likewise, assimilating previous R1 scores with those of other stakeholder groups, reflection and score adjustment was not possible for this individual.

By amending the delivery of R1 scores in both group and one-to-one consultation sessions we identified that people living with dementia critically engaged with the reflection process verbally, and expressed if they wished to change these scores or maintain their original selection. Thus, we established through co-research consultation that verbal administration of R1 scores (for health and social care professionals only), to people living with dementia was the most appropriate approach to deliver R2 of the Delphi survey to this stakeholder group. The choice of health and social care professionals as the single comparison group was based on co-researcher sessions in which people living with dementia felt that their own perspectives would likely differ the most with health and social care professionals. This difference between the perspectives of patients and health and social care professionals is also reflected in the wider literature [28–33].

## Discussion

There is very limited reporting on the participation of people living with dementia in studies using Delphi survey methods [34], and authors have suggested that the method is not appropriate or suitable for people living with dementia [24]. The Delphi design and rating scale selection for the Health Innovation Network South London work did not appear to have involved people living with dementia in design, or adjusted the method to ensure its accessible design. Through team members’: previous experience of working with people living with dementia; dementia training; attending dementia organisation meetings; and through reviewing relevant involvement materials and literature (Alzheimer’s Society; COMET Initiative; DEEP; [9, 17]), we anticipated that consultation with people living with dementia would bring variable approaches and styles to understanding and making use of the information we presented. We were aware that flexible approaches would accommodate individual means of participation, e.g. for those with visuospatial difficulties, word finding or object recognition difficulties. Our COS research design demonstrates that the modification of a Delphi process has enabled the participation of people living with dementia alongside other stakeholder groups. The findings of this Delphi survey, and COS, will be published elsewhere.

We are aware that the participation of people living with dementia in research relies on factors also associated with Patient and Public Involvement more generally. For people living with dementia these factors are especially associated with an individual’s experience and variability of dementia symptoms. Essentially, we describe the need for a flexible, responsive and adaptive approach. Ongoing co-research was planned and facilitated; however, this was constrained through limited study resources anticipated at the time of research design. Our experience has demonstrated that co-research alongside people living with dementia is dependent on a number of factors, which determine the extent to which involvement and influence is achievable. We discuss three such factors below.

### Design of research tools by people living with dementia for use by people living with dementia

A notable feature of our inclusive and accessible approach to co-research was the development of a 3-point rating scale for use by people living with dementia to enable their participation in a modified Delphi method. This scale allowed all outcomes some degree of importance. In so doing we suggest this permitted recognition and respect of the lived experience of people with dementia, through the significant adjustment to a standard research rating scale, in that we did not ask them to classify aspects of their lives as unimportant. Through testing the scale, we observed use across the scale points, reflection by people living with dementia on their choice of scale point, and different selections from carer choice. Thus, confirming the scale as a stakeholder developed and tested method that enabled people living with dementia to participate in complex, sophisticated consensus achieving research methods alongside other key stakeholder groups.

### Allocation of resources to support involvement of people living with dementia in co-research

A main and significant challenge to co-research is time and resource availability. To support people living with dementia in their contributions to research, time is possibly the most important consideration. This is underpinned by the Scottish Dementia Working Group Research Sub-Group Core Principle: ‘keeping to dementia time’. Where researchers do undertake co-research work they need to plan direct contact time with people living with dementia and work at their pace. In our co-research we allowed a minimum of double time for: meeting preparation (e.g. reviewing draft documents); travel and accessing venues; contributing in meetings; participating in activities; and post-meeting follow-up. Significant time was also put into: establishing contacts (with individuals, community or activist organisations); liaison with group organisers; maintaining connections over the study lifetime; participating in organisations’ meetings or activities; developing appropriate materials and lay summaries; facilitating consultation sessions; gaining feedback on developments; and to recognise involvement (e.g. writing acknowledgments and thanks). Furthermore, additional inbuilt time is important to allow flexibility in the moment of involvement. Thus, it is simply not possible to accomplish or value co-research without significant time investment. In the current study, we accommodated considerable slip in timelines in order to achieve consultation on the Delphi survey tool development.

Resources to support our modified Delphi survey development included finance to fund hourly payment rates for co-researcher involvement and for travel and subsistence expenses for both people living with dementia and accompanying care partners INVOLVE 2016 [35] rates were paid for preparation time (where appropriate) and attendance at co-research sessions. Other costs incurred included for materials (colour and large printing), postage, contributions to organisation meetings (e.g. refreshments), and researcher travel costs when people were visited in their homes. While willingness to work in inclusive ways is an important motivator for achieving inclusion, such approaches may not be sufficiently funded or included in study design.

### Training for researchers facilitating dementia co-research

Researcher knowledge and expertise in inclusive research approaches, and skills and awareness in how to support people living with dementia to be involved in research development and design, are necessary to ensure involvement is appropriately supported and achieved [9, 25, 36]. Researchers in the field adhere to ethical research principles as guided through Health Research Authority (UK) Good Clinical Practice and Mental Capacity Act (2005) [37] training. Dementia social scientists are similarly informed through recognised ethical guidelines (for example, see Social Research Association Ethics Guidelines [38] and British Sociological Association Statement of Ethical Practice [39]. In addition to these formal parameters to research practice, researchers need awareness of how dementia symptoms may affect involvement; for example: visuospatial difficulties in reading text; appropriate communication (e.g. not speaking over people when word finding is difficult or providing complex verbal explanations/instructions). In addition, confidence is also important to allow researchers a relaxed demeanour in interacting with people living with dementia, and to be flexible in adjusting arrangements in the moment or seeking appropriate support on behalf of people living with dementia. Investment in training researchers to work in this area is clearly a necessary resource requirement to underpin involvement. For example, our research team attended a whole-programme communication workshop early in the study, which was co-facilitated by people living with dementia and The Neighbourhoods Study partner organisations.

### Support for researchers facilitating dementia co-research

Conducting dementia research in a busy academic environment requires resilient researchers who are supported to deliver high-quality research while working with the challenges we have highlighted. In co-research exchanges, it is not uncommon for academic researchers to be faced with distress, anxiety, grief and declining health, and as such they can undertake emotional labour as part of their work [40–42]. In this context researchers should be supported appropriately in order to maintain their own well-being and perform effectively in their work environment [43]. Our study team members were able to access the The Neighbourhoods Study staff and member well-being service. This service operates as a dedicated Neighbourhoods Study work package and is open to all researchers and staff. The service lead is a clinical psychologist and support is accessed via self-referral for focussed or ongoing support sessions for the duration of The Neighbourhoods Study, in any circumstances of psychological need.

Furthermore, in the longer term, researchers need career opportunities and employment structures to realise professional development. The recognition of the need to support researchers is consistent with the growing emphasis on well-being at work more widely [44–47], and is in line with frameworks such as the ‘mental health core standards’ which highlight the need for employers to monitor the mental health of employees and provide emotional support to employees when needed [48]. To this end, our research team complete a fieldwork template whereby practice or support needs are identified as action points. Within the Neighbourhoods Study, a dedicated, funded work programme provides support for researchers through self-referral to clinical psychologist time for one to one support.

Embedding these features are priorities for future dementia research studies [49–51], as exemplified by the Neighbourhoods Study and recent ESRC-NIHR dementia research initiative 2018. These initiatives have core vision and expectations that co-research is appropriately and effectively resourced, supported at local levels, and strategically developed to build capacity and sustainability. Indeed, funder expectations are that public involvement is an integral element of study design, woven throughout applications rather than shaped in separate sections [52]. Framing co-research as research impact, through extending public engagement and reach of research, may both enable and incentivise researchers to justify resource allocation when inevitably time for engagement activities competes with push for academic outputs for Research Excellence Framework submission. Excluding action research, where participants are involved in activities such as setting the research question, there should be an expectation that funding supports more meaningful co-research through underpinning time and resources, and researcher development. Realistic funding for these areas should be sought in grant proposals such that co-research activity has a visible and sufficiently resourced infrastructure, and its integral value is embedded within individual studies and institutions of academic research [5].

## Conclusion

There is an expectation that researchers conducting studies will facilitate public involvement of the individuals they research. This amounts to involvement that is more than ‘one off’ input (i.e. for feedback on a study tool or measure), but that sees the relations of involvement as a product of social science research and as productive for both contributors; to researched and researchers alike [2].

Inevitably, the extent of involvement is significantly influenced by available resources and funding. Adequate funding, including pre-grant funding, to embed co-research from grant proposal stage into research design and processes is crucial. We emphasise an approach underpinned by flexibility, responsiveness and adaptability to support the involvement of people living with dementia. This approach has facilitated key stakeholder group participation in modified Delphi survey research methods. To be motivated and knowledgeable in this area, and to validate co-research involvement within their roles, researchers need relevant development and training opportunities, access to supervision, and space for professional and personal reflection. Of critical importance for inclusive approaches in dementia research is sufficient dedicated time to develop and maintain working relationships with dementia organisations and individuals over the duration of the study.

## Abbreviations

COINED: CO-research INvolvement and Engagement in Dementia
COMET: Core Outcome Measures in Effectiveness Trials
COS: Core outcome set
DEEP: Dementia Engagement and Empowerment Project
